# Reviewed article: Vale D, Andrade MEC, Dantas NM. Adherence to multiple recommendations of the Dietary Guidelines for the Brazilian Population (Guia Alimentar para a População Brasileira, GAPB) and associated factors among adolescents: a cross-sectional study with data from the National Students’ Health Survey, 2019. Epidemiol Serv Saude. 2025:34;e20240404.

**DOI:** 10.1590/S2237-96222025v34e20240404.b

**Published:** 2025-06-13

**Authors:** Naiara Sperandio

**Affiliations:** Universidade Federal do Rio de Janeiro

**Table:** 

Rating	Excellent	Good	Average	Below average	Poor
Interest		✓			
Quality		✓			
Originality		✓			
Overall		✓			

**Table:** 

Questionnaire	Yes	No	Not applicable
Does the manuscript contain new and significant information to justify publication?	✓		
Does the Abstract (Summary) clearly and accurately describe the content of the article?	✓		
Is the problem significant and concisely stated?	✓		
Are the methods described comprehensively?	✓		
Are the interpretations and conclusions justified by the results?	✓		
Is adequate reference made to other work in the field?	✓		

## Manuscript Structure

Length of article is: Adequate

Number of tables is: Adequate

Number of figures is: Adequate

Please state any conflict(s) of interest that you have in relation to the review of this paper (state “none” if this is not applicable): None

## Comments

Primeiramente, gostaria de parabenizar pelo artigo. A metodologia retrata a robustez com que o estudo foi desenhado e desenvolvido. Apresento algumas questões que são mais esclarecimentos ao realizar a leitura do texto:

-Em relação a introdução a mesma está bem clara e objetiva. Em relação ao objetivo, eu acho que deveria estar descrito da mesma forma como aparece no resumo. A vigilância alimentar e nutricional vem como contribuição que o trabalho traz, não como objetivo direto da pesquisa.

- Métodos:

Em relação a amostra, a partir do momento que houve exclusão de dados, isso não afeta o peso amostral? e consequentemente a expansão da amostra e sua representatividade?

- Em relação as variáveis, a variável dependente foi dicotomizada para as análises bi e multivariada. A pontuação atribuída para criação das categorias dessa variável seguiram algum critério ou foi arbitrário? Eu digo isso, porque, considerar o adolescente que fez entre 0 e 9 como “alguma adesão a múltiplas recomendações do GAPB” e aqueles que fizeram 10 pontos como “ótima adesão a múltiplas

recomendações do GAPB” eu achei uma escala de pontuação estranha. O adolescente que pontuou zero não deveria ser incluído como “alguma adesão”, seria uma ausência de adesão. Houve algum critério de pontuação para construção dessas categorias? Nesse caso a variável original era quantitativa discreta que foi transformada em categórica, seria interessante apresentar os critérios que foram utilizados para criação desse ponto de corte que criou as categorias dessa variável.

Me gerou essa mesma dúvida quando olhei o quadro 1 e para “Múltipla adesão a

recomendações do GAPB com categorias de práticas alimentares recomendadas

possíveis” pontuou 1, quando o consumo de refrigerante era 2 ou menos, porque?

Outra questão, seria interessante deixar claro nesta sessão qual a categoria de referência da variável dependente, isso ficou claro nos resultados.

Discussão:

Os autores discutem, e isso aparece também na conclusão, sobre a avaliação positiva da saúde, no entanto, a percepção de saúde “ruim” também está no modelo múltiplo e não foi discutido possíveis explicações para isso, somente, levou-se a discussão para o resultado positivo de saúde. Importante ressaltar que algumas estratégias elencadas como importantes PNAE e PSE são apenas para escolas públicas, não abrange as escolas privadas. Assim, impulsionar essas ações impacta os adolescentes de escolas públicas, sendo importante o planejamento de ações para as privadas também.

Em relação a potencialidades do trabalho, reforço minha pergunta inicial sobre a representatividade. Ainda pode-se considerar representativo mesmo com exclusão de indivíduos da amostra inicial do IBGE? 

